# Integrating Functional Response and Target Binding for Mechanism-Centered Drug Screening by High-Mass MALDI-MS

**DOI:** 10.1021/acscentsci.5c01944

**Published:** 2026-01-27

**Authors:** Congrui Tan, Yu Gao, Marcus Buggert, Yuye Zhou, Renato Zenobi

**Affiliations:** † Department of Chemistry and Applied Biosciences, Swiss Federal Institute of Technology (ETH), CH-8093 Zürich, Switzerland.; § Department of Medicine Huddinge, Center for Infectious Medicine, 27106 Karolinska Institutet, 14152 Stockholm, Sweden; ¶ School of Engineering Sciences in Chemistry, Biotechnology and Health, Department of Chemistry, Division of Applied Physical Chemistry, Analytical Chemistry, 426712 KTH Royal Institute of Technology, SE-10044 Stockholm, Sweden

## Abstract

Early stage drug discovery is limited by the disjunction of function and binding assays, creating an information gap that leads to the high failure rate in hit advancement. This limitation is particularly pronounced for protein–protein interactions, whose large and shallow interfaces make it difficult to distinguish hits mechanistically. To address this, we developed a cross-linking matrix-assisted laser desorption/ionization mass spectrometry (MALDI-MS) platform that integrates biochemical functional response and target binding in a single assay, thereby generating a multidimensional pharmacological profile. Using the SARS-CoV-2 RBD–ACE2 interaction and a set of 17 drug candidates for a proof-of-concept study, the platform revealed a clear difference between two inhibitors that appeared indistinguishable in conventional functional assays: one showed stronger affinity and preferential ACE2 binding, while the other showed weaker and less specific binding. These mechanistic differences were consistent with the results of a cellular antiviral assay, in which only the high-affinity inhibitor improved cell viability. This work presents a mechanism-centered, rapid screening strategy that provides early multiparameter insight, enables rational selection of high-quality leads for challenging drug targets, and is compatible with high-throughput formats.

## Graphical abstract

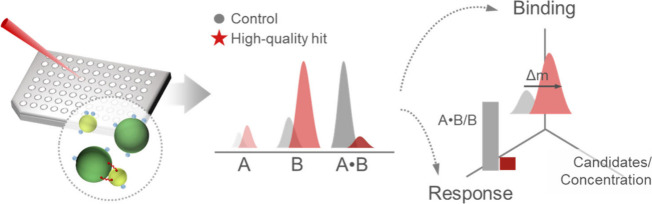

## Introduction

A critical challenge in traditional experimental drug discovery is the high failure rate of candidate compounds, often due to fragmented data collected during early screening.^1,2^ Protein–protein interactions (PPIs) are especially compelling therapeutic targets but remain among the most difficult to assay.^3^ Unlike ligand-protein systems with well-defined binding pockets, PPI interfaces are broad, shallow, and featureless, making rational design particularly challenging.^4,5^ Therefore, strategies that can provide a more multidimensional and mechanistically relevant description of compound behavior are urgently needed.

The field of drug discovery has advanced along two distinct paths. Function-first strategies (phenotypic screening), such as high-throughput biochemical assays including AlphaScreen and cell-based reporter assays, rapidly identify compounds with biological effects. However, they provide little direct information about target binding, which often leads to high downstream failure.^6,7^ Binding-first strategies (target-based screening), exemplified by DNA-encoded libraries and fragment-based drug discovery, ensure binding from the start. Yet, without functional context, these approaches also require extensive secondary validation.^8,9^

Despite their differences, both approaches face the same fundamental limitation: an information gap. Each yields only part of the pharmacological picture, forcing reliance on additional assays to connect target binding with functional response. This blind spot has long limited the efficiency of drug discovery and addressing it is a necessary step toward improving success rates.^10^ In a context such as the COVID-19 pandemic, this gap hinders the rapid identification of effective therapeutics.^11,12^ Although AI-driven drug discovery increasingly bridges this gap in silico, experimental workflows are still required, but lack a unified solution.^13,14^

Here we present an integrated screening platform designed to bridge this gap. Mass spectrometry (MS) is widely used in drug discovery for its speed and sensitivity. Native electrospray ionization mass spectrometry (ESI-MS) can in principle probe intact noncovalent complexes, but necessitates ESI-compatible, volatile buffers.^15^ Moreover, heterogeneous proteins and protein complexes can yield very congested spectra with many charge states, requiring charge reduction/deconvolution. In contrast, matrix-assisted laser desorption/ionization (MALDI-MS) exhibits a higher salt tolerance, and spectra consist predominantly of singly charged signals, which simplifies multicomponent analysis.^16,17^ MALDI is also compatible with high-throughput formats. Leveraging these advantages and building on our previous work to stabilize protein–protein complexes by cross-linking in solution prior to analyzing them with high-mass MALDI-MS,^17–19^ we developed a workflow that simultaneously quantifies the biochemical functional response (as a normalized peak-area ratio of the complex to the corresponding free partner protein) and target binding (as compound-induced shifts in the target-protein peak mass relative to the control) in a single assay. Using the interaction between the severe acute respiratory syndrome coronavirus 2 (SARS-CoV-2) receptor-binding domain (RBD) and angiotensin-converting enzyme 2 (ACE2) as a model, we demonstrate how this approach bridges the gap between function-first and binding-first paradigms, enabling a more comprehensive evaluation of candidate inhibitors.

## Results and Discussion

### A Platform for Integrated Function–Binding Profiling

To address the information gap between function-only and binding-only screening, we developed a cross-linking high-mass MALDI-MS platform that integrates both dimensions in a single assay. The workflow consists of three steps (Figure 1a), and is demonstrated here for the case of the RBD–ACE2 interaction. First, recombinant RBD and ACE2 proteins are incubated with test compounds to reach equilibrium, and the resulting RBD•ACE2 complex is stabilized by chemical cross-linking. Next, the samples are deposited on a 384-well plate using a sandwich spotting method and analyzed by high-mass MALDI-MS. Quantitative workflow descriptors are summarized in Supporting Information (S5.3, Table S2). Mature end-to-end MALDI-TOF automation has been established for high-throughput screening,^16^ therefore, scaling our workflow to such pipelines would only require inserting a parallel microplate “mix-and-incubate” cross-linker step prior to spotting.

**Figure 1:**
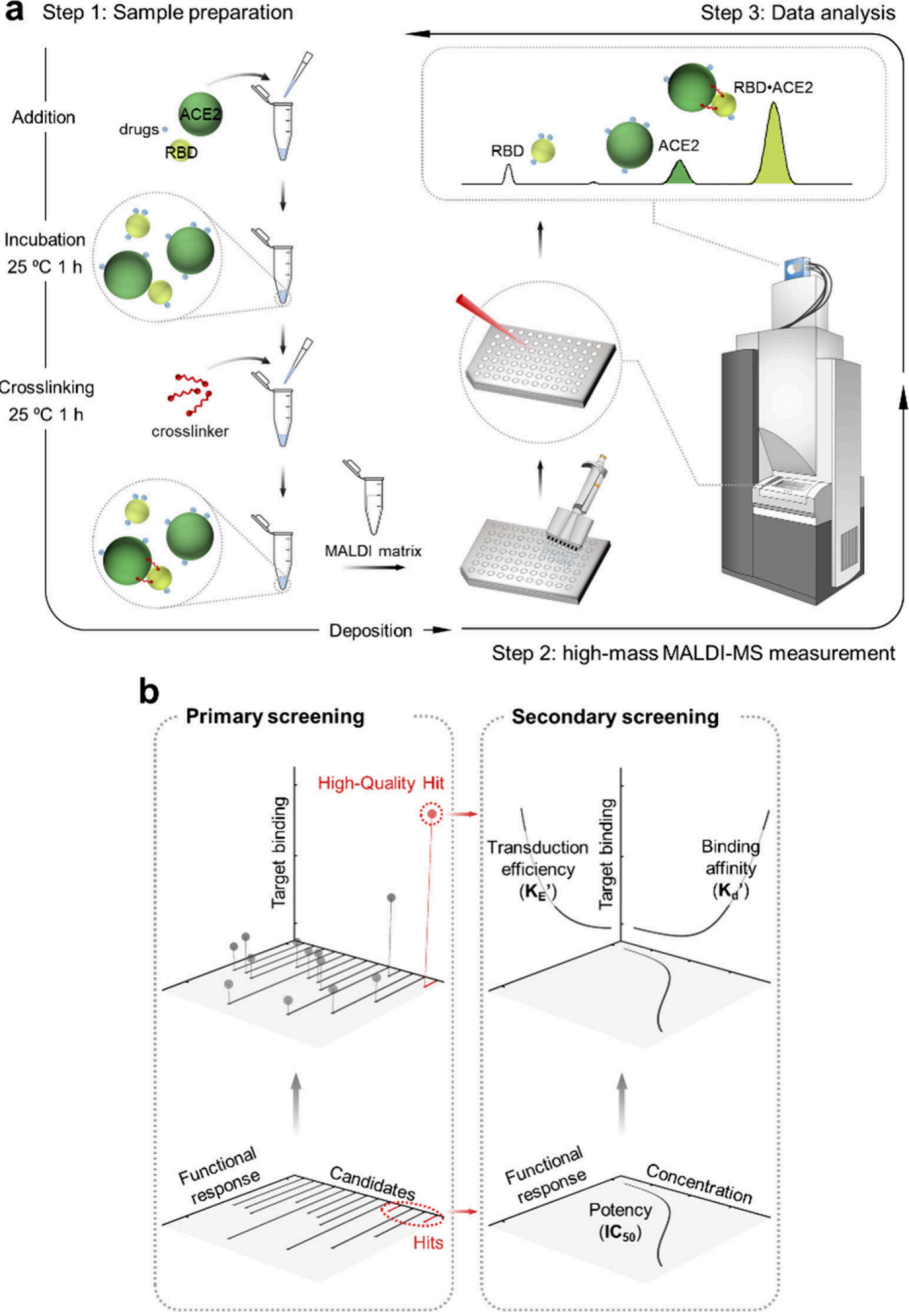
Integrated function–binding profiling platform. (a) The three-step workflow: sample preparation, high-mass MALDI-MS analysis, and dual-parameter data analysis. (b) The dual readout elevates screening from a conventional 2D function-only plane to a 3D function-binding space. This enables the selection of high-quality hits in primary screening and pharmacological profiling (IC_50_, *K*_d_′, *K*_E_′) in secondary screening.

Finally, the spectra are processed to generate two orthogonal readouts: the first is a biochemical functional response, reported as relative PPI activity (area ratio of the RBD•ACE2 complex to free ACE2, eq S1), and its complement, the inhibition effect (100%-relative PPI activity, eq S2). The second readout is target binding, calculated from compound-induced mass shifts of the target proteins (eqs S3 and S4). Together, these readouts provide a multidimensional profile that enables direct comparison and selection of compounds. This dual-readout capability elevates screening from the conventional 2D function-only plane to a 3D function-binding space (Figure 1b).

Our platform operates in two sequential stages (Figure 1b). Primary screening in the 3D space enables selection of high-quality hits. Secondary screening with dose–response experiments further resolves the space into a pharmacological profile consisting of potency (IC_50_, eq S5), apparent binding affinity (*K*_d_′, eq S6), and apparent transduction efficiency (*K*_E_′, eq S7), as defined, for example, in chapters 3 and 4 of *Rang & Dale’s Pharmacology*.^20^ The equations and the curve-fitting procedures based on the Black and Leff operational model are provided in the Supporting Information (Section 1, S1.3). In this framework, target binding derived from mass shifts serves as a proxy for receptor occupancy, yielding apparent parameters (*K*_d_′, *K*_E_′). We therefore describe this dual readout as function-binding, which integrates a biochemical functional response (relative PPI activity or inhibition effect) with information on target binding (MS-derived proxy for occupancy).

### Functional Profiling Identifies Two Indistinguishable Inhibitors

We began by screening a library of 17 FDA-approved drugs (Table S1), selected from literature reports describing their activity against the RBD–ACE2 interaction.^21–36^ In the primary screening, drugs were evaluated at a single concentration of 50 μM. As shown in representative mass spectra (Figure 2a, top), effective inhibitors reduced the signal of the RBD•ACE2 complex relative to free ACE2. The free RBD peak was excluded from quantitative analysis because its signal was often found to be attenuated and less reproducible in drug-treated samples. Conversely, under 1:1 PPI conditions, the RBD•ACE2 complex/ACE2 area ratio directly reports the fraction of ACE2 engaged in complex and therefore provides a robust inhibition metric for screening.

**Figure 2:**
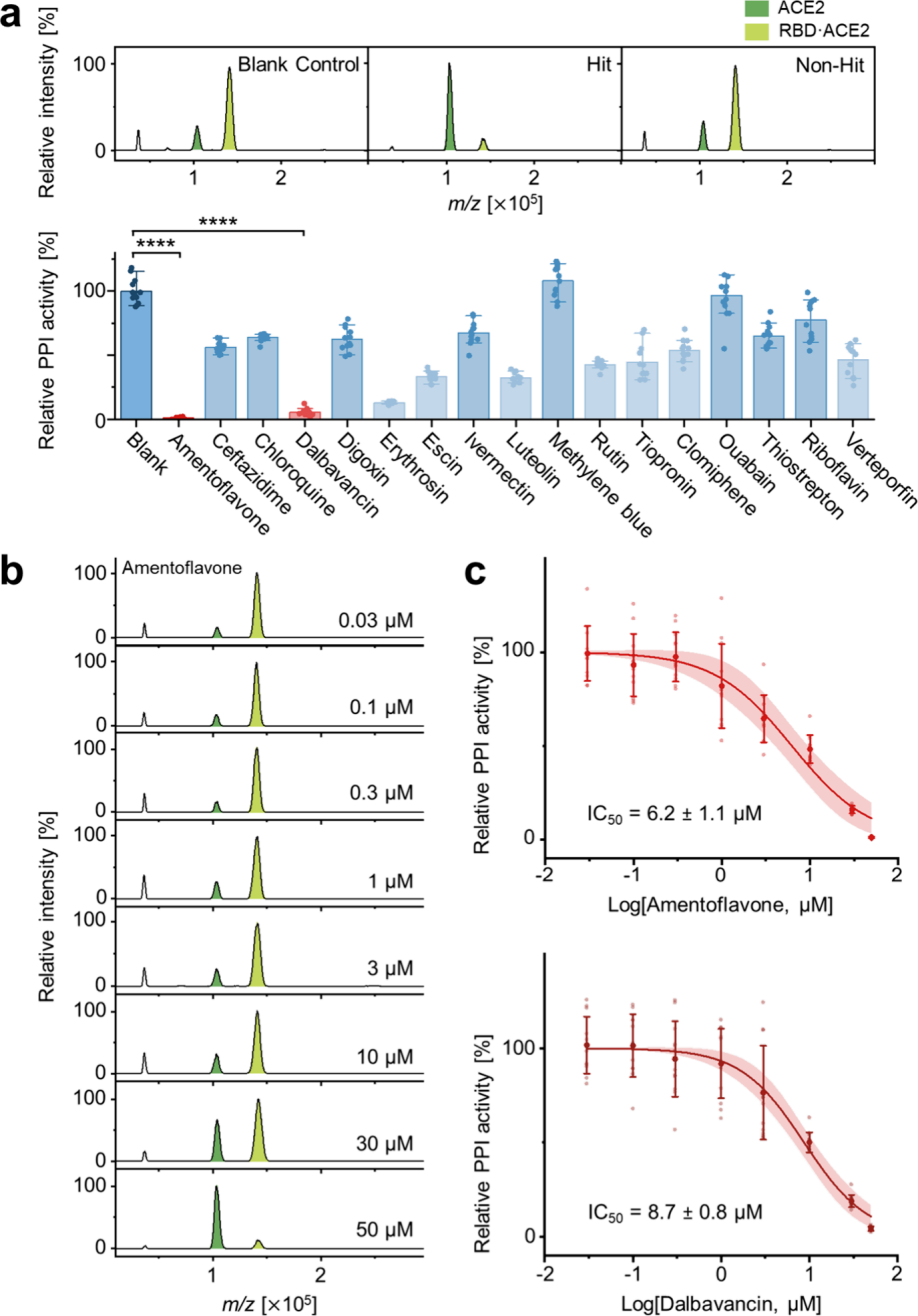
Two drugs remain indistinguishable in the 2D function plane. (a) Primary screening results for 17 drug candidates. Representative mass spectra (top) illustrate inhibition of the RBD–ACE2 interaction, with mass spectra for all 17 candidates in Figure S1. Relative PPI activity analysis (bottom) identified amentoflavone and dalbavancin as the most potent hits (****p < 0.001, observed *p* < 10^–7^, One-way ANOVA with a Tukey’s test). Amentoflavone versus dalbavancin was not significant (*q* = 1.55, *p* = 0.9998). (b) Secondary screening by dose–response analysis. Mass spectra at varying concentrations showed concentration-dependent inhibition for amentoflavone, with the corresponding spectra for dalbavancin provided in Figure S2. (c) The resulting dose–response curves yielded nearly identical IC_50_ values for amentoflavone and dalbavancin.

A one-way analysis of variance (ANOVA) with a post hoc Tukey’s test for relative PPI activity across all drugs revealed that amentoflavone and dalbavancin were the top performers (**** *p* < 0.001, observed *p* < 10^–7^,) (Figure 2a, bottom). A direct comparison between amentoflavone and dalbavancin did not show any significant difference (*q* = 1.55, p = 0.9998). Mass spectra for all tested drugs are provided in Figure S1.

To further compare these two hits, we performed a dose–response analysis. Both amentoflavone and dalbavancin showed a clear concentration-dependent inhibition, as seen in the spectra (Figure 2b, Figure S2). Plotting the relative PPI activity against drug concentration yielded classic dose–response curves (Figure 2c), with IC_50_ values of 6.2 ± 1.1 μM for amentoflavone and 8.7 ± 0.8 μM for dalbavancin.

Thus, even after progressing from initial hit identification to potency determination within the 2D function plane, amentoflavone and dalbavancin remained essentially indistinguishable, making rational selection impossible based on functional metrics alone.

### Function–Binding Profiling Reveals Mechanistic Divergence

To resolve the ambiguity between amentoflavone and dalbavancin, we re-examined the primary screening data by incorporating the binding readout acquired in parallel with the function response. In the RBD–ACE2 system, plotting the inhibition effect (100%-relative PPI activity, eq S2) against overall binding across all 17 drugs showed a positive correlation (Figure 3a). A multivariate analysis of variance (MANOVA) confirmed that the hits (dalbavancin, amentoflavone) and nonhits formed statistically distinct populations (Wilks’ Lambda, *p* < 0.0001). Consistent with this, a one-way ANOVA with a Tukey’s test on the total binding showed that both dalbavancin and amentoflavone bound significantly more than nonhits (*p* < 0.0001), with dalbavancin showing higher binding than amentoflavone (*p* < 0.0001). This validated that both drugs are high-quality hits, combining strong inhibition effect with significant target binding.

**Figure 3:**
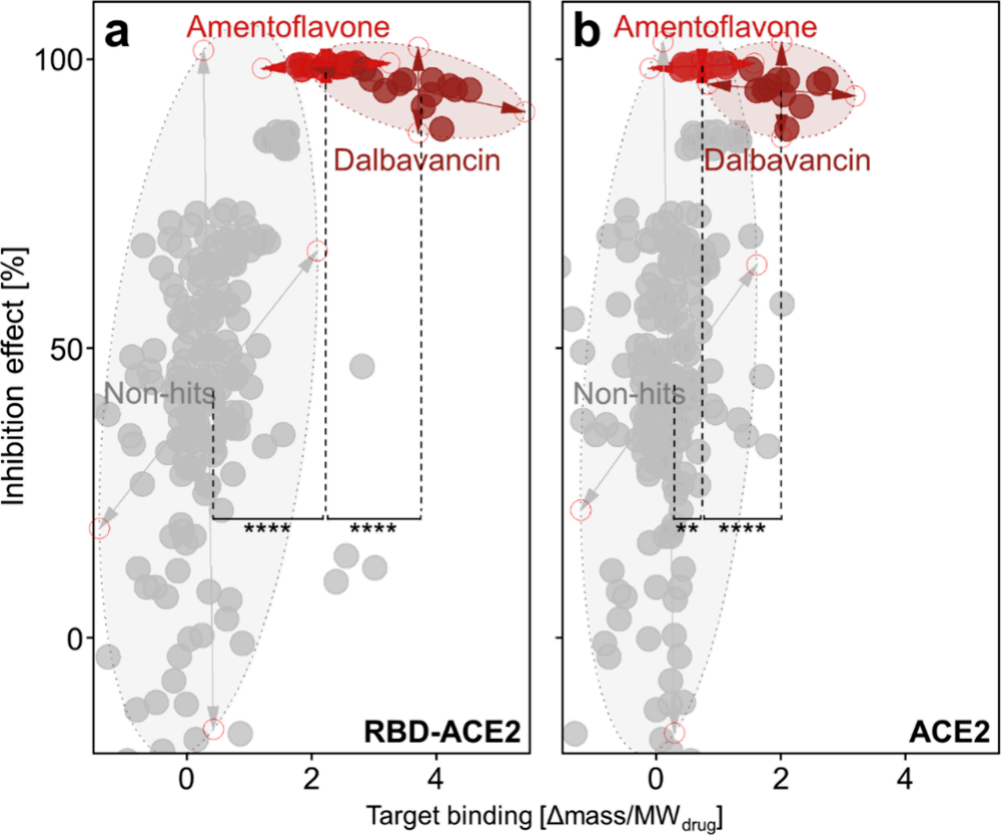
Re-interpretation of primary screening data with target binding. (a) RBD–ACE2 system binding analysis. Hits (red) and nonhits (gray) were statistically distinct (MANOVA, Wilks’ λ = 0.4704, *p* < 0.0001). One-way ANOVA with a Tukey’s test on total binding showed that dalbavancin and amentoflavone both exceeded nonhits (*p* < 0.0001), with dalbavancin exceeding amentoflavone (*p* < 0.0001). (b) ACE2-specific binding analysis. Dalbavancin remained strongly separated from nonhits (*p* < 0.0001) and exceeded amentoflavone (*p* < 0.0001), whereas amentoflavone showed less pronounced separation from nonhits (*p* < 0.01), highlighting preferential ACE2 engagement of dalbavancin.

To investigate mechanistic differences, we examined ACE2-specific binding (Figure 3b). In this dimension, dalbavancin remained strongly separated from nonhits (*p* < 0.0001) and exceeded amentoflavone (*p* < 0.0001), whereas amentoflavone showed less pronounced separation from nonhits (*p* < 0.01). Compared with total binding, the ACE2-specific analysis reduced the distinction of amentoflavone from nonhits while preserving the separation of dalbavancin, thereby amplifying the contrast between two hits. Taken together with a significant MANOVA when ACE2 binding replaced total binding (*p* < 0.0001), these results support preferential ACE2 binding by dalbavancin and weaker ACE2 binding by amentoflavone.

To quantify these differences, we constructed 3D pharmacological profiles using dose–response data fitted with the Black and Leff operational model (Figure 4). While potency (IC_50_) was similar (6.2 ± 1.1 μM for amentoflavone and 8.7 ± 0.8 μM for dalbavancin), their apparent binding affinity (*K*_d_′) to the system differed by an order of magnitude, with dalbavancin (13.2 ± 2.6 μM) showing significantly stronger affinity than amentoflavone (140.0 ± 72.9 μM). Transduction efficiency (*K*_E_′) provided additional detail: amentoflavone (0.4 ± 0.1 μM) showed slightly higher efficiency in converting binding into inhibition than dalbavancin (1.0 ± 0.2 μM). However, in the context of hit selection, the much stronger affinity and higher ACE2 binding of dalbavancin are the key factors, outweighing the modest efficiency advantage of amentoflavone.

**Figure 4:**
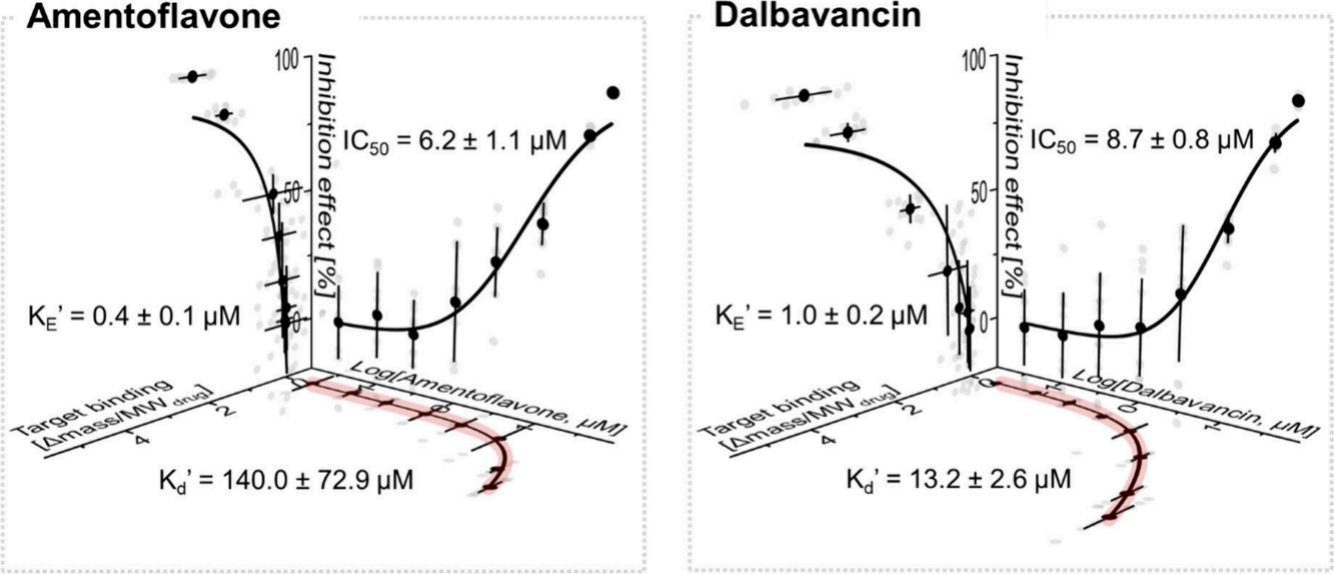
3D pharmacological profiling from secondary screening data. Dose–response data for amentoflavone and dalbavancin fitted with the Black and Leff operational model. The resulting pharmacological profiles yielded potency (IC_50_), binding affinity (*K*_d_′), and transduction efficiency (*K*_E_′). Dalbavancin showed stronger affinity and higher ACE2 binding, while amentoflavone showed slightly higher transduction efficiency.

This integrated, multiparameter analysis enabled rational differentiation of hits that could not be achieved with functional screening alone. Binding trends were consistent with validation by native ESI-MS (Figure S3).

### Orthogonal Cellular Validation Confirms the MALDI-MS-Based Prediction

To test whether the differential ACE2 binding revealed by our MALDI-MS platform is functionally relevant, we implemented a selective cellular antiviral assay. A key feature of this design was a wash step following compound pretreatment, which removed unbound or weakly associated drugs before viral infection. This specifically probed the cellular functional response of inhibitors with stable target binding. Based on our MALDI-MS results, we hypothesized that dalbavancin, with high ACE2 binding, would be retained more on the cell surface and improve cell viability, whereas amentoflavone, with lower binding, would be retained less and therefore show a limited effect.

The experimental outcome was consistent with this prediction (Figure 5). At 24 h post infection, no drug had a significant effect. At 48 h, dalbavancin-treated cells showed a dose-dependent increase in viability relative to the untreated control (*p < 0.05 at 25 μM; **p = 0.0011 at 50 μM). In contrast, amentoflavone failed to improve cell viability at either concentration, and viability even showed a slight decrease at higher concentrations. To examine whether this decrease was due to cytotoxicity, we performed a dedicated cytotoxicity test. Amentoflavone was nontoxic at 24 h, but minor cytotoxicity emerged at 100 μM after 48 h (Figure S4).

**Figure 5:**
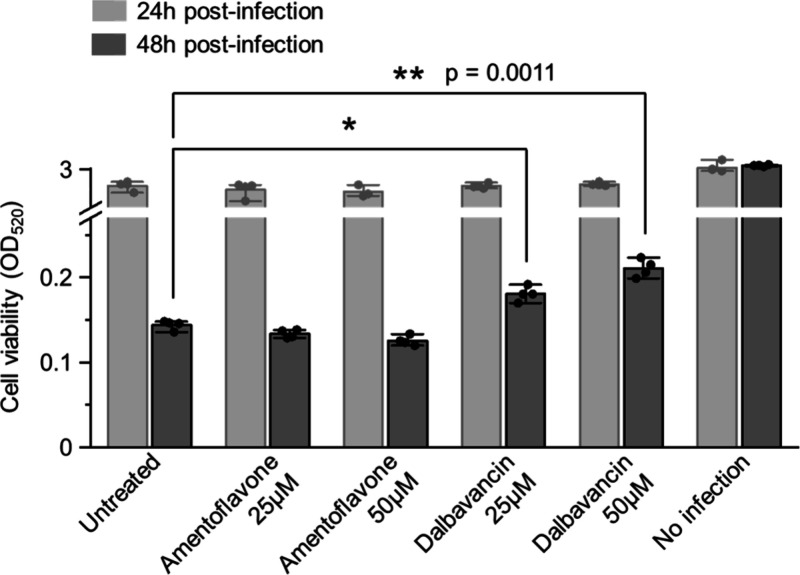
Dalbavancin, but not amentoflavone, improves cell viability during SARS-CoV-2 infection. Vero E6 cells were pretreated with the indicated drug concentrations, washed, and then infected with SARS-CoV-2. Cell viability was measured at 24 and 48 h post infection. Data are mean ± SD from four replicates (*n* = 4). Statistical significance relative to the 48 h untreated control was assessed by one-way ANOVA with a Tukey’s test. Significance is indicated as *p < 0.05 and **p < 0.01.

These findings provide direct biological validation of the results obtained with our screening platform. The performance of dalbavancin under selective wash conditions was confirmed as a high-quality, on-target lead. In contrast, the lack of a cellular functional response for amentoflavone, together with its cytotoxicity at higher concentrations, illustrates the risk of advancing drugs that are potent but mechanistically ambiguous. These results demonstrate that the ability of our integrated platform to deliver early mechanistic insight is predictive of cellular function and essential for distinguishing true therapeutic candidates from misleading drugs.

### Advantages, Limitations, and Future Applications of the MALDI-MS Platform

The most distinctive advantage of our MALDI-MS platform is that it unifies biochemical functional response and target binding within a single, high-throughput compatible assay. This dual-readout capability not only bridges the information gap between function-first and binding-first paradigms but also enables early, mechanism-based selection of high-quality hits at scale. We envision this platform to be used for post-primary screening, where the number of candidates has already been narrowed down and resources are available for replicates and detailed function-binding profiling. Such selection is critical for efficient allocation of resources and for avoiding the advancement of compounds that are potent but mechanistically flawed.

Other advanced biophysical techniques, such as high-throughput surface plasmon resonance (HT-SPR), provide valuable kinetic data.^37–40^ Our MALDI-MS platform, however, offers a complementary advantage. As a homogeneous solution-phase assay, it evaluates interactions in a state closer to the native environment and avoids artifacts from protein immobilization. The 384-well plate format is compatible with automated liquid handling and spotting systems, making the platform scalable for high-throughput screening. In addition, the use of MALDI-MS offers a practical advantage in its tolerance to physiological buffers. Unlike many biophysical methods that require extensive buffer exchange, this approach is compatible with physiological salt concentrations, enabling the study of protein–protein interaction systems that are sensitive to buffer composition under conditions that better mimic the cellular environment.

While our high-mass MALDI-MS readout requires cross-linking, this does not materially constrain its utility under defined operating conditions. We use an amine-reactive NHS-ester cross-linker as a postequilibration locking step to preserve preformed PPI complexes, rather than to drive complex formation.^41^ Operationally, this adds only a simple “mix-and-incubate” step. Because primary amines are common in proteins, NHS-ester locking is generally transferable across PPIs, provided that solvent-accessible amines are available at or near the interaction interface; moving to a new PPI system only requires a one-time compatibility check (S5.2). Moreover, since NHS-esters can be quenched by free amines, the reaction is best implemented in amine-free, MS-compatible buffers. Finally, cross-linking does not create artifacts: prior controls on the same RBD–ACE2 system have shown that the cross-linker stabilizes only complexes that are already present in solution rather than creating nonspecific complexes.^19^

It should be noted that the target binding and derived pharmacological parameters (*K*_d_′ and *K*_E_′) reported in this work are apparent parameters, and thus semiquantitative. A potential limitation is that the cross-linking step stabilizes the protein–protein complex but not the noncovalent drug compound-protein interaction. This creates a risk of laser-induced dissociation during the MALDI process, which may underestimate absolute binding. However, because higher-affinity interactions are more stable, they are more likely to survive desorption and ionization. As a result, while absolute values may be underestimated, the platform reliably preserves the relative rank ordering of candidates. The reliability of binding trends measured by our MALDI-MS-based method was further supported using native electrospray ionization mass spectrometry (ESI-MS), a softer ionization technique (see Figure S3 for details). For the purpose of future high-throughput screening and hit selection, this relative quantification is well suited.

Looking forward, the platform can be applied beyond inhibitor discovery. Its ability to detect any perturbation of a protein–protein complex makes it suitable for identifying molecular modulators, including stabilizers and allosteric activators. This extends its utility from drug discovery into chemical biology, where it can be used to explore diverse mechanisms of molecular regulation.

## Conclusions

This work demonstrates an integrated, high-throughput compatible screening platform that unifies the function-first and binding-first paradigms by simultaneously acquiring biochemical functional response and target binding in a single assay. The value of this platform is illustrated by the case of amentoflavone and dalbavancin inhibiting the SARS-CoV-2 RBD–ACE2 interaction. Although both appeared similar potent in a conventional biochemical functional screen, the platform distinguished dalbavancin as a high-quality lead, characterized by strong affinity and an on-target profile predictive of cellular functional response. In contrast, amentoflavone was identified as a high-risk candidate due to weak affinity and weaker ACE2 engagement, consistent with its lack of cellular activity.

In conclusion, this work advances a more rational, mechanism-centric paradigm for screening. By providing high-throughput and multidimensional data early in discovery, such platforms have the potential to reduce the failure rate, improve resource efficiency, and accelerate the development of new medicines. This capability is especially critical in urgent contexts such as the COVID-19 pandemic, and for rapid response to future emerging infectious disease threats.

## Supplementary Material



## Data Availability

The original data used in this publication are made available in a curated data archive at ETH Zurich (https://www.research-collection.ethz.ch) under the DOI 10.3929/ethz-b-000716698.

## Notes

Table S1. The 17 FDA-approved drug candidates selected for screening; Table S2. Workflow descriptors for automation compatibility and scalability; Figure S1. Complete MALDI mass spectra from the primary screening of 17 drug candidates; Figure S2. Dose-dependent MALDI mass spectra for dalbavancin at concentrations ranging from 0.03 μM to 50 μM; Figure S3. Comparison of mass shift results with ESI and MALDI source; Figure S4. Cytotoxicity testing of amentoflavone (PDF)
