# Supplementary material for: Integrating Functional Response and Target Binding for Mechanism-Centered Drug Screening by High-Mass MALDI-MS
Source: ACS Cent Sci. 2026 Jan 27;12(3):316–23. doi: 10.1021/acscentsci.5c01944 (PMC13022716; doi:10.1021/acscentsci.5c01944)
Supplement: Supplementary file 1 [file oc5c01944_si_001.pdf]

Supporting Information

Integrating Functional Response and Target Binding for Mechanism-Centered Drug Screening by High-Mass MALDI-MS

Congrui Tan,‡,† Yu Gao,‡,§ Marcus Buggert,,§ Yuye Zhou,*,¶,†, and Renato Zenobi,*,†*

† Department of Chemistry and Applied Biosciences, Swiss Federal Institute of Technology (ETH), CH-8093 Zürich, Switzerland.

§ Department of Medicine Huddinge, Center for Infectious Medicine, Karolinska Institutet, 14152 Stockholm, Sweden

¶ School of Engineering Sciences in Chemistry, Biotechnology and Health, Department of Chemistry, Division of Applied Physical Chemistry, Analytical Chemistry, KTH Royal Institute of Technology, SE-10044 Stockholm, Sweden.

Corresponding author emails:

marcus.buggert@ki.se

yuyzhou@ethz.ch

renato.zenobi@org.chem.ethz.ch

Supporting Information

Section 1: Data Analysis and Curve Fitting Equations S3
S1.1: Function Calculation S3
S1.2: Target Binding Calculation S4
S1.3: Black & Leff Operational Model and Curve Fitting S4
S1.3.1: Potency Profile (Concentration vs. Function) S4
S1.3.2: Affinity Profile (Concentration vs. Target Binding) S5
S1.3.3: Transduction Profile (Target Binding vs. Function) S5
Section 2: Supplementary Screening Results and Figures S7
Section 3: ESI-MS Validation of the MALDI-MS Binding Assay S10
Section 4: Assessment of Amentoflavone Cytotoxicity S11
Section 5: Methods & Materials S12
S5.1: Materials S12
S5.2: Drug-RBD-ACE2 interaction and crosslinking S13
S5.3: HM-MALDI-TOF MS analysis S13
S5.4: Virus Preparation S15
S5.5: Virus Infection and Cell Viability Assay S15
S5.6: ESI-MS analysis S15
REFERENCES S16

Section 1: Data Analysis and Curve Fitting Equations

This section provides the detailed equations used for the calculation of function and target binding from the raw mass spectrometry data, and for the curve fitting procedures used to derive the pharmacological parameters.

S1.1 Function Calculation

The function of each test compound was evaluated based on its ability to inhibit the formation of the RBD•ACE2 protein complex. This was quantified from the MALDI mass spectra by first calculating the Relative Activity.

The Relative Activity of the RBD•ACE2 interaction was calculated as the ratio of the peak area of the protein complex to that of free ACE2 in a drug-treated sample, normalized to the no-drug control (proteins in buffer only), as shown in Equation (S1).

$$Realitve\ activity = \frac{\left[\frac{Area_{RBD\bullet ACE2}}{Area_{ACE2}}\right]_{sample}}{\left[\frac{Area_{RBD\bullet ACE2}}{Area_{ACE2}}\right]_{control}} * 100\% \qquad (S1)$$

Where:

$Area_{RBD\bullet ACE2}$ is the peak area of the RBD•ACE2 complex in the sample.

$Area_{ACE2}$ is the peak area of free ACE2 in the sample.

For subsequent analyses and plotting, the Relative Activity was converted into Inhibition Effect using Equation (S2).

$$Inhibition\ effect = 100\% - Realitve\ PPI\ activity \qquad (S2)$$

S1.2 Target Binding Calculation

The Target Binding, a semi-quantitative parameter reflecting the extent of drug-protein engagement, was calculated from the mass shift of the target proteins induced by the compound.

The total Target Binding on the entire protein system (RBD and ACE2) was calculated by summing the mass shifts of both proteins in a drug-treated sample relative to the no-drug control, and then dividing by the molecular weight of the test drug, as shown in Equation (S3). Similarly, the Target Binding specific to the ACE2 protein was calculated using only the mass shift of ACE2, as shown in Equation (S4).

$$Target\ Binding_{[RBD-ACE2]} = \frac{[Mass_{RBD} + Mass_{ACE2}]_{sample} - [Mass_{RBD} + Mass_{ACE2}]_{control}}{Mw_{drug}} \quad (S3)$$

$$Target\ Binding_{[ACE2]} = \frac{[Mass_{ACE2}]_{sample} - [Mass_{ACE2}]_{control}}{Mw_{drug}} \quad (S4)$$

Where:

$Mass_{protein}$ is the measured mass of the specified protein in the sample.

MW_{drug} is the molecular weight of the test drug.

S1.3 Black & Leff Operational Model and Curve Fitting

Dose-response data were analyzed to derive key pharmacological parameters using equations derived from the Black & Leff operational model. All curve fitting was performed using OriginPro (OriginLab Corporation, Northampton, MA).

S1.3.1 Potency Profile (Concentration vs. Function)

The half-maximal inhibitory concentration (IC50) was determined by fitting the dose-response data of Inhibition Effect versus drug concentration to a four-parameter logistic equation, as shown in Equation (5).

$$y = \frac{1}{1 + 10^{(\log IC_{50} - x) * HillSlope}} \tag{S5}$$

Where:

y is the Inhibition Effect, normalized to a maximum of 1.

x is the logarithm of the drug concentration.

IC50 is the concentration that produces 50% of the maximal inhibition.

HillSlope is the Hill coefficient of the curve.

S1.3.2 Affinity Profile (Concentration vs. Target Binding)

The apparent dissociation constant (K_d') was determined by fitting the Target Binding versus drug concentration data to a one-site binding model that includes a term for non-specific binding, as shown in Equation (S6).

$$y = \frac{B_{max} * 10^x}{K'_d + 10^x} + k * 10^x \tag{S6}$$

Where:

y is the Total Target Binding = Target Binding$_{[RBD\text{-}ACE2]}$, equation S3

x is the logarithm of the drug concentration.

B_{max} is the maximum specific binding.

K_d' is the apparent dissociation constant.

k is the slope for non-specific binding.

S1.3.3 Transduction Profile (Target Binding vs. Function)

The apparent transduction coefficient (K_E'), which describes the efficiency of converting target engagement into an inhibitory effect, was determined by fitting Inhibition Effect versus Target Binding data to a hyperbolic function, as shown in Equation (S7). Transduction Profile (Target Binding vs. Function):

$$y = \frac{E_{max} * x}{K'_E + x} \quad \text{(S7)}$$

Where:

y is the Inhibition Effect from equation S2.

x is the Target Binding.

E_{max} is the maximum possible effect.

K_E' is the apparent transduction coefficient.

Section 2: Supplementary Screening Results and Figures

Table S1. The 17 FDA-approved drug candidates selected for screening.

Name	CAS number	MW
Amentoflavone	1617-53-4	538.46
Ceftazidime	72558-82-8	546.57
Chloroquine	54-05-7	319.87
Clomiphene	50-41-9	598.1
Dalbavancin	171500-79-1	1816.69
Digoxin	20830-75-5	781.05
Erythrosin	16423-68-0	879.86
Escin	6805-41-0	884
Ivermectin	70288-86-7	875.09
Luteolin	491-70-3	286.24
Methylene blue	61-73-4	319.86
Ouabain	11018-89-6	728.77
Riboflavin	83-88-5	376.36
Rutin	207671-50-9	610.153
Thiostrepton	1393-48-2	1664.89
Tiopronin	1953-02-2	163.19
verteporfin	129497-78-5	718.8

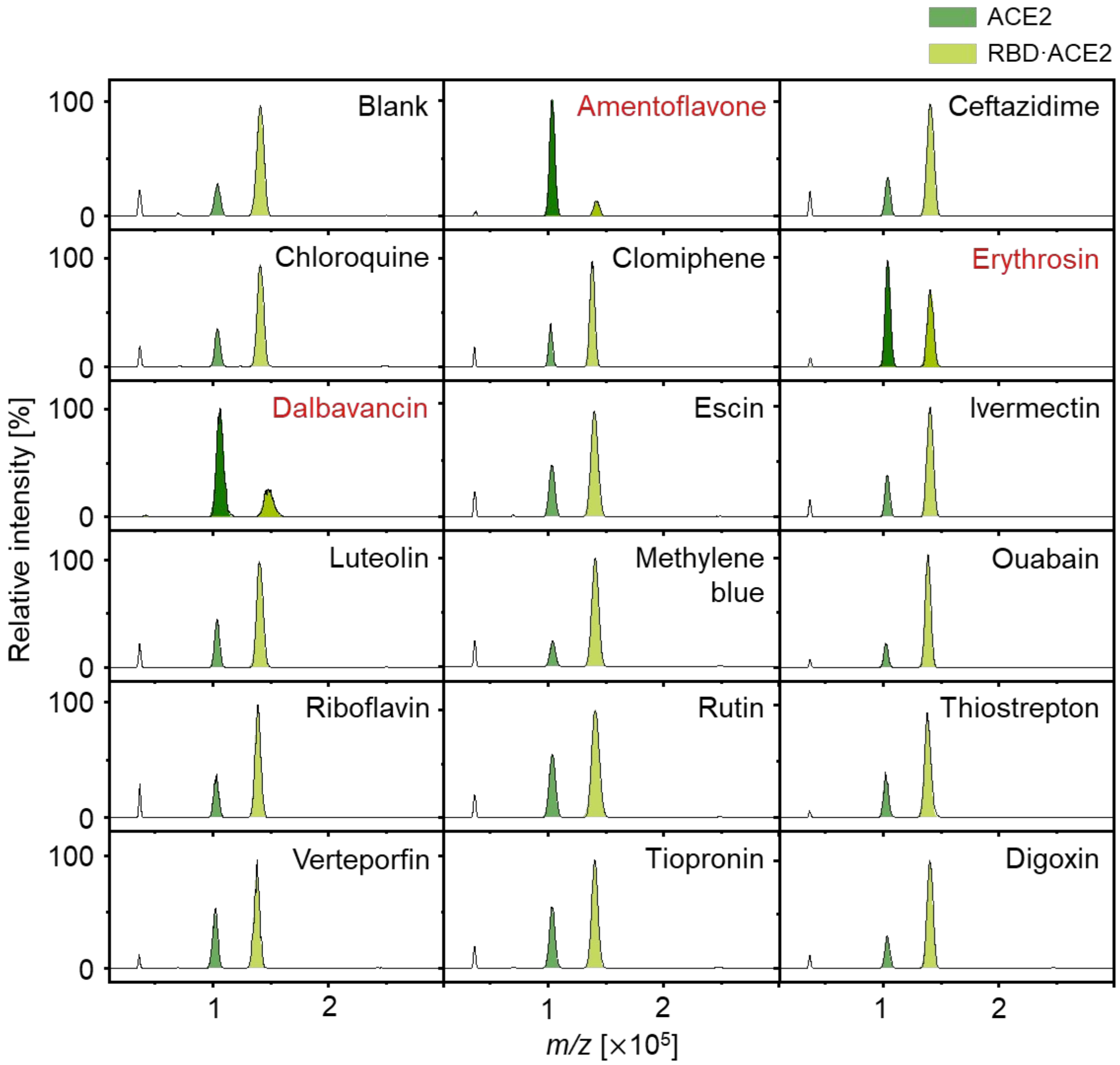

Figure S1. Complete MALDI mass spectra from the primary screening of 17 drug candidates. This figure shows the raw data used to calculate the Relative Activity presented in the main text, Figure 2a. Spectra for the control (no drug), a representative non-hit, and the two top hits (amentoflavone and dalbavancin) are highlighted.

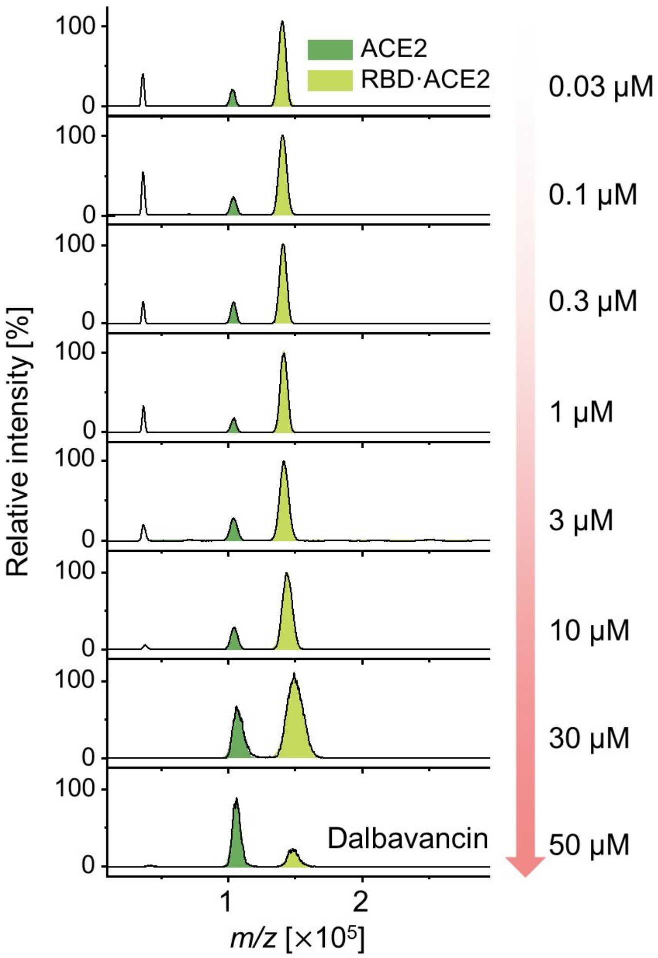

Figure S2. Dose-dependent MALDI mass spectra for dalbavancin at concentrations ranging from 0.03 µM to 50 µM. These data were used to generate the dose-response curves shown in the main text, Figure 2b.

Section 3: ESI-MS Validation of the MALDI-MS Binding Assay

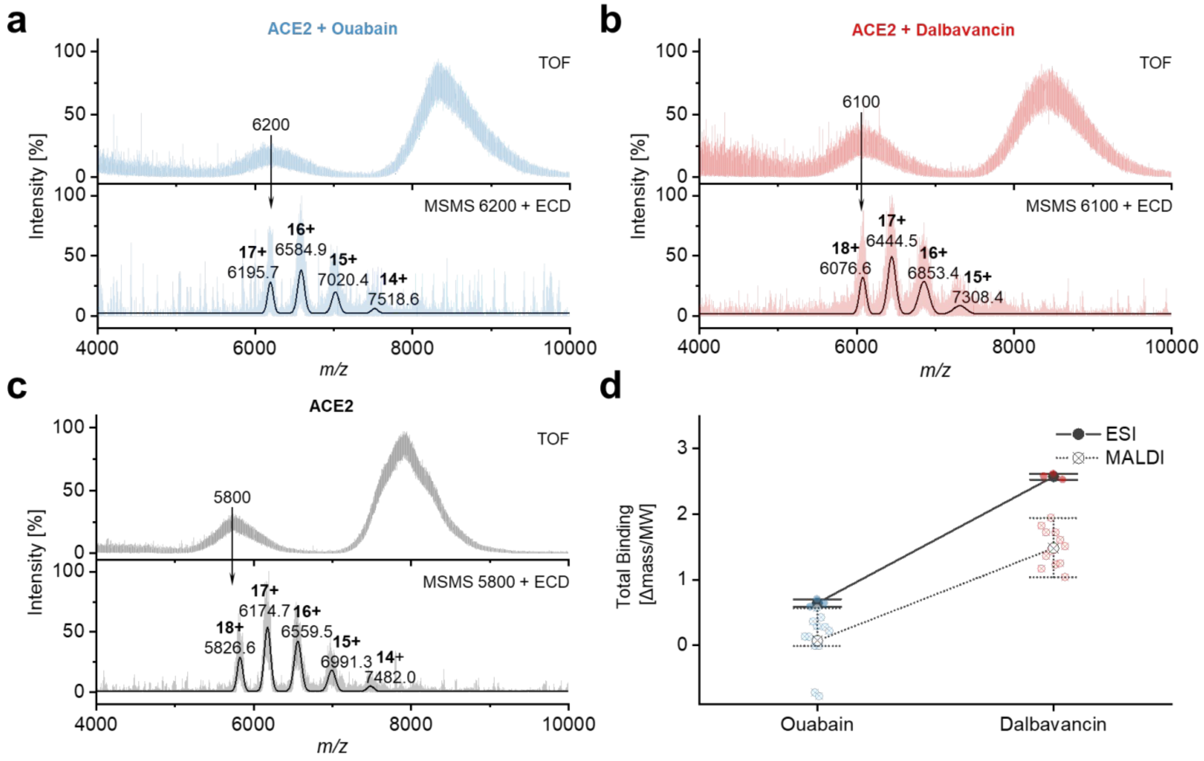

Figure S3. Comparison of mass shift results with ESI and MALDI source. (a) Mass spectrum of dalbavancin-incubated ACE2 protein. (b) Mass spectrum of ouabain-incubated ACE2 protein. (c) Mass spectrum of ACE2 protein. (d) ACE2 binding results measured with different ionization sources: the same trend of dalbavancin showing stronger binding to ACE2 while ouabain showing weaker binding to ACE2 measured with both ESI and MALDI sources.

Section 4: Assessment of Amentoflavone Cytotoxicity

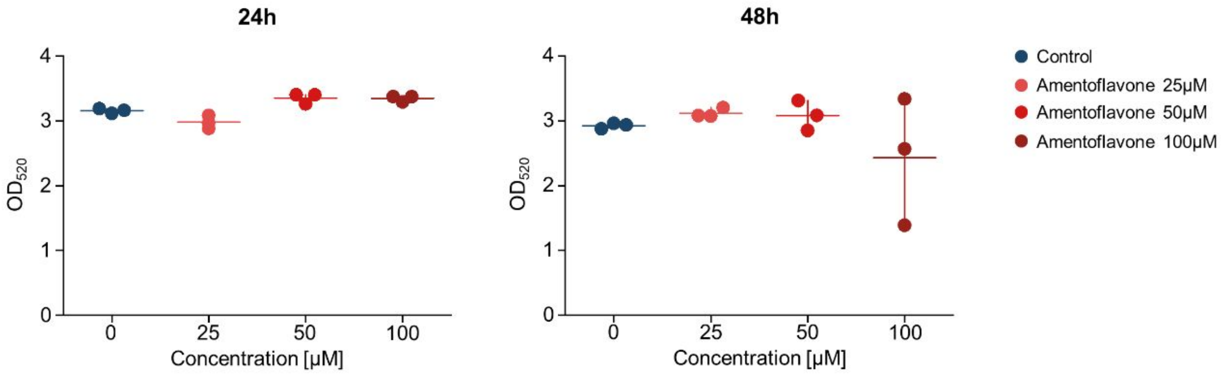

Figure S4. Cytotoxicity testing of amentoflavone. At 24 hours post-infection, no cytotoxicity was observed across varying amentoflavone concentrations. However, at 48 hours post-infection, cells treated with high concentrations of amentoflavone exhibited minor cytotoxicity.

Section 5: Methods & Materials

S5.1 Materials

SARS-CoV-2 spike RBD (Omicron B.1.1.529, 40592-V08H121) and human ACE2 (10108-H08H) were purchased from Sino Biological (Germany) and received in lyophilized form from sterile PBS (phosphate buffered saline, 1x, pH 7.4). PEGylated bis(sulfosuccinimidyl)suberate (BS(PEG)9) was purchased from Thermo Fisher Scientific (USA). Trifluoroacetic acid (TFA) and acetonitrile (ACN) were bought from Sigma-Aldrich, sinapinic acid (SA) from Tokyo Chemical Industry (Eschborn), and dimethyl sulfoxide (DMSO) from VWR chemicals. For the drug compounds employed, the following catalog numbers and suppliers were utilized: rutin (132390050, Thermo Scientific Chemicals), ceftazidime (C2225, Tokyo Chemical Industry), dalbavancin (SML2378, Sigma-Aldrich), luteolin (72511, Supelco), amentoflavone (AB253890, abcr GmbH), escin (E1378, Sigma-Aldrich), erythrosin extra bluish (45690, Sigma-Aldrich), methylene blue (A1402, AppliChem), ivermectin (I8898, Sigma-Aldrich), digoxin (AB133920, abcr GmbH), tiopronin (T2614, Tokyo Chemical Industry), chloroquine (AB436023, abcr GmbH), ouabain octahydrate (O3125, Sigma-Aldrich), verteporfin (17334, Adipogen AG), thiostrepton from streptomyces azureus (BIT1003, Apollo Scientific Ltd), clomiphene citrate (16087, Adipogen AG), and riboflavin (BIR0613, Apollo Scientific Ltd). All reagents and solvents were used as received, without further purification.

The RBD and ACE2 proteins were reconstituted utilizing Milli Q water (H2O) to yield a stock concentration of 0.5 mg/mL and 2 mg/mL, respectively. The concentrations of these proteins were verified by employing a UV NanoDrop 2000/2000c spectrophotometer (Thermo Fisher Scientific, Wilmington, USA). To prevent potential deterioration resulting from multiple freezing and thawing cycles, 5 μL aliquots of RBD and ACE2 were stored at −20 ˚C. Each drug was weighed by an analytical balance (XS205DU, Mettler Toledo, Switzerland) and subsequently dissolved in DMSO, achieving a stock concentration of 10 mM. The drug stock solutions obtained were then aliquoted to 10-50 μL and stored at -20 ˚C. For the subsequent drug binding experiments, both the

RBD and ACE2 protein solutions with or without the drugs present, were further diluted with H2O.

S5.2 Drug-RBD-ACE2 interaction and crosslinking

The RBD and ACE2 proteins were both diluted to a fixed final concentration of 1 μM within the RBD-ACE2 interaction system. We found in separate control experiments that at this concentration ACE2 dimerization can be minimized. The total salt concentration in the final solution was ~ 20 mM. In the primary screening stage, different drugs at the same final concentration of 50 μM were introduced to the fixed RBD-ACE2 interaction system. The drug concentration of 50 μM was chosen because it provides a sufficient signal despite salt interference and allows for the differentiation of drug inhibition abilities. In the secondary screening stage, titration experiments were conducted, involving the addition of various final concentrations of the screened drugs: 0.03 μM, 0.1 μM, 0.3 μM, 1 μM, 3 μM, 10 μM, 30 μM, and 50 μM. All dilutions were performed using Milli Q water. For each experiment, an additional blank sample was prepared, consisting of the same fixed RBD-ACE2 interaction system without any drugs. After the mixing of drugs and proteins, an incubation of 1 hour at 25 °C was maintained to ensure the equilibrium state of the drug-RBD-ACE2 interaction system. The formed RBD•ACE2 complex was stabilized by crosslinking with BS(PEG)$_9$ (final concentration of 1 mM) at 25 °C for 1 hour before HM-MALDI-TOF MS analysis. The selection of the crosslinker was predicated on the spacer distance between the lysine (K) residues in the RBD•ACE2 complex interface based on previous work[1].

To adapt the locking step to a new PPI, one needs to perform a one-time, minimal check guided by interface geometry: (1) identify solvent-accessible primary amines near the interaction interface using an available structure and estimate representative inter-amine distances; (2) select an NHS-ester crosslinker spacer length that matches these distances as a starting condition. This is typically required only once for a new PPI system.

S5.3 HM-MALDI-TOF MS analysis

Sinapinic acid was dissolved in a mixture of acetonitrile and Milli Q water containing 0.1% trifluoroacetic acid (TFA) at ratios of 30/70 (v/v) and 50/50 (v/v) under sonication for 10 minutes, resulting in saturated matrix solutions (called SA30 and SA50 below). Samples following the incubation and crosslinking processes were applied onto a MALDI plate employing a sandwich method: 0.4 μL of SA30 was deposited as the basal layer on the MALDI plate; after the drying of the basal layer, 2 consecutive layers of 0.5 μL of the sample were applied step by step; the top layer of 0.5 μL SA50 was deposited once the previous layers were dried. Each reaction underwent three replicates, and a total of four spots were obtained for each replicate (12 spots in total). Mass spectra were acquired using a MALDI-TOF/TOF mass spectrometer (model 4800 plus, AB Sciex, Darmstadt, Germany) equipped with a high-mass detector (HM2, CovalX AG, Zurich, Switzerland). Desorption/ionization was done by a Nd:YAG laser (355 nm) at a laser intensity of 7000 a.u., and detection was performed in the linear positive mode. The high voltages 1 and 2 of the high-mass detector were set to -3.5 kV and -20.0 kV, respectively. A random walk pattern across the entire spot was conducted, and 500 shots were accumulated for each individual spectrum.

Data analysis (Step 3) was conducted using a home-developed program based on MATLAB, which aims to identify both the ACE2 and RBD•ACE2 complex peaks. After peak identification, it can calculate the peak area ratio of RBD•ACE2 to ACE2 for each spectrum.

Table S2. Workflow descriptors for automation compatibility and scalability

Module	Descriptor	Value in this work
Reaction setup	Protein concentrations	RBD = 1 μM<br>ACE2 = 1 μM
Reaction setup	Drug concentration (primary screen)	50 μM
Reaction setup	Incubation to equilibrium	1 h at 25 °C
Crosslinking	Crosslinker, concentration	$BS(PEG)_9$, 1 mM final
Crosslinking	Crosslinking time/temperature	1 h at 25 °C
Deposition	Spotting format	Sandwich spotting on MALDI plate
Deposition	Per-spot volumes/layers	0.4 μL SA30 (base)<br>+ 2 × 0.5 μL sample<br>+ 0.5 μL SA50 (top)
Replication	Replicates and spots	3 replicates; 4 spots/replicate (12 spots total)
Acquisition	Sampling pattern	Random walk across entire spot
Acquisition	Shots per spectrum	500 shots accumulated per spectrum
Data processing	Automated peak identification and readout	MATLAB program identifies peaks and computes peak area ratio
Cadence[2]	Representative time per spectrum	~8 s per spectrum reported for the same HM-MALDI platform

S5.4 Virus Preparation

The TCID50 was calculated using the Spearman & Kärber algorithm, as described in Hierholzer & Killington (1996), Virology Methods Manual, p. 374.

S5.5 Virus Infection and Cell Viability Assay

200,000 Vero E6 cells were seeded into 96-well plates. After 4 hours, based on previous experimental data, cells were treated with varying concentrations of the drugs amentoflavone (25 μM and 50 μM) and dalbavancin (25 μM and 50 μM) to block the ACE2 receptor. The control group was treated with medium as a blank control. After 2 hours, the drugs were removed, and the

cells were washed twice with PBS. Subsequently, cells were infected with SARS-CoV-2 at an MOI of 0.1. At 4 hours post-infection, the virus was removed, and the cells were washed twice with PBS. The same drug treatments were re-applied to block the ACE2 receptor, with the control group receiving medium. At 24 and 48 hours post-infection, cell viability was assessed using the Sulforhodamine B Protein Assay (SRP assay) following the method described by Vichai and Kirtikara[3].

S5.6 ESI-MS analysis

ACE2 protein samples were prepared from the stock solution and used size exclusion chromatography in 100 mM AmAc on a fast protein liquid chromatography system (Äkta micro, GE Healthcare Life Sciences, Marlborough, MA, USA) with a Superdex 200 increase column (10 × 300 mm, Cytiva) to remove salt content, followed by concentration using spin columns, resulting in a final protein concentration of 5.6 μM (confirmed with a UV NanoDrop 2000/2000c spectrophotometer (Thermo Fisher Scientific, Wilmington, USA)) in 100 mM ammonium acetate (pH 7.4). A validation experiment was conducted with three samples: ACE2, ACE2 with dalbavancin, and ACE2 with ouabain. The final concentration of ACE2 was 2.8 μM, and the final concentrations of dalbavancin and ouabain were both 50 μM. All dilutions were carried out using Milli-Q water. The same incubation conditions at 25 °C for 1 hour were maintained for comparison with HM-MALDI-MS analysis.

The SELECT SERIES Cyclic ion mobility-mass spectrometry (Cyclic-IMS, Waters, Wilmslow, U.K.) coupled with an electron capture dissociation (ECD) cell in the post-IMS region was used to measure the samples. Each sample solution was sprayed from a borosilicate capillary of ~1 μm I.D. (B100-75-10, Sutter Instruments, California), prepared using a micropipette puller (P-1000, Sutter Instruments), and fitted with a platinum wire. All samples were sprayed in positive mode. The essential parameters of the mass spectrometer were as follows: capillary voltage 1.3 kV, sampling cone 20 V, source offset 30 V, source temperature 28 °C, trap collision energy 5 V,

transfer collision energy 5 V. Mass spectra were first acquired in MS mode to scan the mass range for ACE2 monomer samples. Due to the heterogeneity caused by glycosylation, different charge states of ACE2 samples were congested. To resolve charge states, the mass showing the highest intensity was selected for each sample for further MSMS analysis with electron capture charge reduction. The rhenium filament was turned on for charge reduction and the current was 2.1 A. In this way, the charge states of ACE2 samples can be resolved and the mass can be calculated.

REFERENCES

1. Zhou, Y.;Tan, C.;Zenobi, R. Rapid Profiling of the Glycosylation Effects on the Binding of SARS-CoV-2 Spike Protein to Angiotensin-Converting Enzyme 2 Using MALDI-MS with High Mass Detection. *Analytical Chemistry* **2024,** *96*, 1898-1905.
2. Wu, N.;Olechwier, A. M.;Brunner, C.;Edwards, P. C.;Tsai, C.-J.;Tate, C. G.;Schertler, G. F. X.;Schneider, G.;Deupi, X.;Zenobi, R.;Ma, P. High-mass MALDI-MS unravels ligand-mediated G protein–coupling selectivity to GPCRs. *Proceedings of the National Academy of Sciences* **2021,** *118*, e2024146118.
3. Vichai, V.;Kirtikara, K. Sulforhodamine B colorimetric assay for cytotoxicity screening. *Nature Protocols* **2006,** *1*, 1112-1116.
